# Potential Association of Isolated γ-Glutamyltransferase Elevation with Incident Ischemic Heart Disease in Lean Koreans

**DOI:** 10.3390/jpm12121966

**Published:** 2022-11-28

**Authors:** Yumin Sung, Yong-Jae Lee, Dong-Hyuk Jung, Byoungjin Park

**Affiliations:** 1 Department of Family Medicine, Yongin Severance Hospital, Yongin-si 16995, Republic of Korea; 2 Department of Family Medicine, Gangnam Severance Hospital, Seoul 06273, Republic of Korea

**Keywords:** γ-glutamyltransferase elevation, cardiometabolic risk, cohort study, ischemic heart disease

## Abstract

Isolated elevation of γ-glutamyltransferase (GGT), a microsomal membrane-bound protein, is commonly observed in non-obese Koreans without diabetes, and its clinical implications are not well-known. Therefore, we aimed to investigate the longitudinal effect of isolated GGT on the incidence of ischemic heart disease (IHD) risk in a large cohort of lean non-diabetic Koreans. Data were obtained from the Health Risk Assessment Study (HERAS) and Korea Health Insurance Review and Assessment (HIRA) datasets. The participants were divided into four groups according to the GGT quartile after the exclusion of those participants with diabetes, a body mass index (BMI) ≥ 25 kg/m^2^, alanine aminotransferase (ALT) ≥ 40 IU/L, and aspartate aminotransferase (AST)/ALT > 1.5, as well as those positive for hepatitis B surface antigen or hepatitis C antibody. We prospectively assessed the hazard ratios (HRs) with 95% confidence intervals (CIs) for IHD using multivariate Cox proportional hazard regression models over a 50-month period. During the follow-up period, 183 individuals (1.85%) developed IHD. After setting the lowest GGT quartile as a reference group, the HRs of IHD for GGT quartiles 2–4 were 1.66 (95% CI 0.95–2.89), 1.82 (95% CI 1.05–3.16), and 1.98 (95% CI 1.12–3.50), respectively, after adjusting for age, sex, body mass index, smoking status, alcohol consumption, physical activity, mean arterial blood pressure, fasting plasma glucose, and dyslipidemia. An isolated high GGT may be an additional measure for assessing and managing future IHD risks among lean Koreans without diabetes.

## 1. Introduction

Serum γ-glutamyltransferase (GGT) concentrations are usually elevated with other liver enzymes, such as aspartate aminotransferase (AST), alanine aminotransferase (ALT), and alkaline phosphatase (ALP). However, in non-obese Koreans, it is not uncommon for GGT elevation to occur without an increase in other liver enzymes.

GGT elevation has long been understood in connection with hepatobiliary disease, but it has been found to exist in most cells, including in the liver, the kidneys, the pancreas, the spleen, and the brain [1,2]. GGT is an enzyme on the external surface of cellular membranes and plays an essential role in maintaining the physiological concentration of cellular glutathione, which maintains the redox balance and detoxifies chemicals [3,4].

At the cellular level, elevated GGT levels can indirectly reflect an increased oxidative stress burden and are directly involved in the pathophysiology of the atherosclerotic process [5]. From a clinical perspective, GGT levels are closely related to increased adiposity or alcohol consumption [6]. Furthermore, higher GGT levels have been associated with diabetes, metabolic syndrome, and cardiovascular disease (CVD) in many clinical contexts [7,8,9].

To date, the cardiometabolic implications of isolated GGT elevation have not been well-known in Koreans without obesity. Thus, this study aimed to examine the longitudinal association between isolated GGT and IHD incidence in a lean Korean population without diabetes.

## 2. Materials and Methods

### 2.1. Data Collection

The current study was derived from a Korean population-based cohort whose data were extracted from the Health Risk Assessment Study (HERAS) and Korea Health Insurance Review and Assessment Service (HIRA) datasets. A cohort in the urban areas of South Korea was enrolled to explore surrogate indicators for IHD through the collection of metabolic parameters and health-related behaviors [10,11]. Initially, 20,530 ambulatory individuals in the HERAS, who voluntarily participated in a physical examination, were included in the baseline survey from November 2006 to June 2010. All the participants in the cohort were connected to the HIRA, the universal coverage system in Korea, and the study outcomes were entirely based on ICD.

The subjects meeting any of the following criteria were excluded: previously diagnosed with IHD, ischemic stroke, or diabetes mellitus (including individuals with a fasting plasma glucose level ≥ 126 mg/dL) (n = 1590); under 20 years of age; fulfilling the diagnostic criteria for general obesity in Korea: body mass index (BMI) ≥ 25 kg/m^2^ [12]; alanine aminotransferase (ALT) ≥ 40 IU/L [13]; aspartate aminotransferase (AST)/ALT > 1.5 (to minimize the inclusion of alcoholic steatohepatitis) [14]; positive for hepatitis B surface antigen or hepatitis C antibody; the presence of liver cirrhosis; and the current use of aspirin (n = 9046). Lean non-diabetic participants were defined as individuals with BMI < 25 kg/m^2^ and without diabetes mellitus before or at baseline.

**Figure 1:**
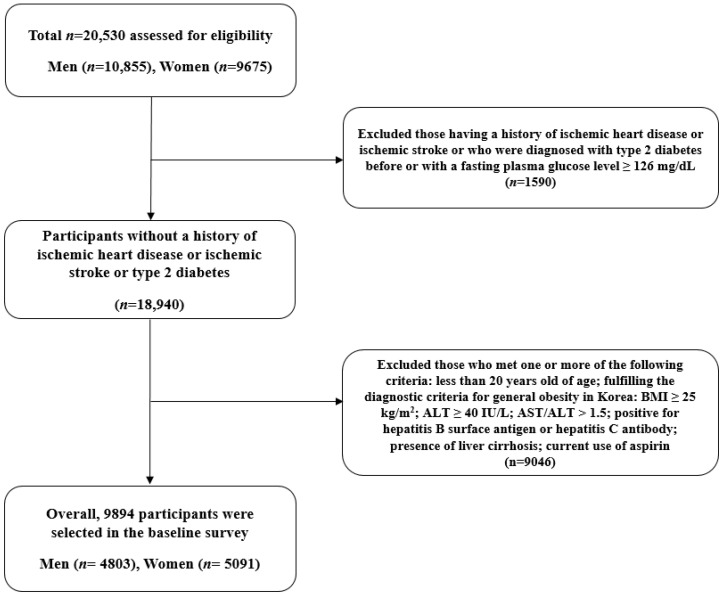
Flowchart for the selection of study participants. BMI, body mass index; AST, aspartate aminotransferase; ALT, alanine aminotransferase.

After the application of the exclusion criteria, 9894 individuals (4803 men and 5091 women) were included in the final analysis (Figure 1). The materials and methodology of the HERAS–HIRA dataset were described in detail in previous studies [10,11]. We divided the entire population according to GGT quartiles as follows: Q1 ≤ 14 (≤25th percentile); Q2: 15–19 (26 to 50th percentile); Q3: 20–29 (51 to 75th percentile); and Q4 ≥ 30 (≥76th percentile). For the subgroup analysis, serum GGT levels were additionally categorized into quartiles by men and women in the same way. We calculated the HS index (HSI) score as follows: HSI score = 8 × ALT/AST ratio + BMI (kg/m^2^) (+2 for women) [15]. Dyslipidemia was defined as total cholesterol ≥240 mg/dL, triglycerides ≥150 mg/dL, high-density lipoprotein (HDL) cholesterol <40 mg/dL for men and <50 mg/dL for women, or the use of lipid-lowering medication. Prediabetes was defined as fasting plasma glucose levels between 100 mg/dL and 126 mg/dL. The study protocol was approved by the Institutional Review Board of the Yonsei University College of Medicine. The data of the participants were provided anonymously after they signed an informed consent form.

### 2.2. Study End Point

The outcomes were incidental IHD, angina pectoris (ICD-10 code I20), or acute myocardial infarction (ICD-10 code I21), which were assessed over the 50 months since the initial enrollment by linking each unique 13-digit identification number to the HIRA database.

### 2.3. Statistical Analysis

We used box plots and the Kolmogorov–Smirnov test to evaluate the distribution of the variables. The GGT values with skewed distributions were categorized into quartiles as follows: Q1 (≤14), Q2 (15–19), Q3 (20–29), and Q4 (≥30). The baseline characteristics were compared according to the GGT quartiles using a chi-squared test and analysis of variance (ANOVA) for categorical and continuous variables, respectively. Age- and sex-adjusted survival curves were used to estimate the cumulative incidence of IHD for each group. We utilized a Cox proportional hazard regression model to assess the hazard ratios (HRs) and 95% confidence intervals (CIs) for the incidence of IHD after adjusting for age, sex, body mass index, smoking status, alcohol consumption, physical activity, mean arterial blood pressure, fasting plasma glucose, and dyslipidemia. We also calculated the HRs and CIs for the incidence of IHD according to sex-based GGT quartiles in the same way, removing sex in the covariates. An ex post power calculation was also performed: for an HR of 1.5, the calculated power was >0.999; for an HR of 2.0, the calculated power was >0.999. All the statistical analyses were performed using the SAS software (version 9.4; SAS Institute Inc., Cary, NC, USA). Statistical significance was set at *p* < 0.05.

## 3. Results

**Figure 2:**
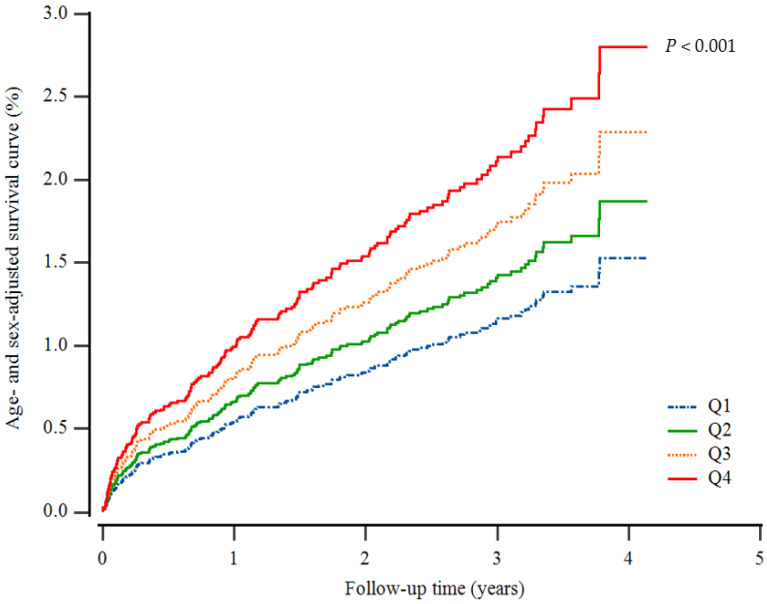
Cox regression survival curve for the incidence of ischemic heart disease.

Table 1 shows the baseline characteristics of the study population (n = 9894; 4803 men and 5091 women) according to GGT quartiles. The mean age and BMI of the study population were 44.9 ± 10.4 years and 22.0 ± 1.9 kg/m^2^, respectively. The mean AST and ALT concentrations were 19.5 ± 4.9 IU/L and 19.0 ± 6.9 IU/L, and the mean HSI score was 30.8 ± 2.8. The mean BMI, mean arterial pressure, total cholesterol, and log-transformed C-reactive protein levels were the highest, while the mean HDL-C levels were the lowest in the fourth GGT quartile group. The greatest proportion of current smokers, alcohol consumption, and hypertension were members of the highest GGT quartile. The higher GGT group exhibited a significantly elevated cumulative incidence of IHD up to 50 months after adjusting for age and sex (Figure 2).

Table 2 shows the results of the multivariate Cox proportional hazards regression analysis for the prediction of IHD according to the GGT quartiles. A total of 183 individuals (1.85%, 183/9894) developed IHD during the study period. The incidence rate (per 1000 people–years) of IHD proportionally increased as the GGT quartile increased. Compared with the referent GGT quartile, the HRs of the new-onset IHD were 1.66 (95% CI 0.95–2.89), 1.82 (95% CI 1.05–3.16), and 1.98 (95% CI 1.12–3.50) for the second, third, and fourth GGT quartiles, respectively, after adjusting for age, sex, body mass index, smoking status, alcohol intake, physical activity, mean arterial blood pressure, fasting plasma glucose, and dyslipidemia.

For the subgroup analysis by sex-specific quartile, the GGT values were categorized into Q1 (≤19), Q2 (20–26), Q3 (27–38), and Q4 (≥39.0) in men and Q1 (≤12), Q2 (13–15), Q3 (16–20), and Q4 (≥21) in women. Compared with men, women showed higher HRs for the risk of the incidence of IHD in the highest quartile (HR (95% CI) = 1.23 (0.71–2.14) and 2.86 (1.19–6.87), respectively) (Table 3).

## 4. Discussion

This large cohort study of lean non-diabetic Koreans found that isolated high GGT was positively associated with IHD incidence, independent of health behaviors and major cardiometabolic parameters. In the subgroup analysis by sex-specific quartile, these associations between GGT and the incidence of IHD were more prominent in women than in men. Moreover, the HRs of overt IHD in women significantly increased according to the GGT quartiles; contrastingly, those in men did not exhibit significant trends.

Some possible explanations for the observed association deserve consideration. In increased oxidative stress, the breakdown of glutathione by GGT in extracellular spaces is activated, and the production of cysteinylglycine dipeptide is promoted, which works as a more potent reducing agent than glutathione. In this process, superoxide and hydrogen peroxide formation is enabled, concomitantly causing lipid peroxidation and proinflammatory responses [5,16,17]. These reactions can occur within atherosclerotic plaques, promoting atherosclerotic progression with plaque vulnerability [18,19,20]. This study also found that the log-transformed CRP concentration increased as the GGT quartile increased. According to the data from the Korea National Health and Nutrition Examination Survey (KNHANES), GGT was closely related to oxidative stress and inflammatory burden [3]. Studies on the relationship between GGT and CVD are limited among relatively lean Asians. A well-designed, prospective study was conducted in Japan, where 2724 men and 4122 women were followed up for 9.6 years to evaluate CVD motility. The participants with increased GGT levels showed an increase in the HR for CVD-related death to 2.88 in women but showed no significant results in men [21]. The prevalence of drinkers in East Asian women was very low compared with that in men, and when corrected for alcohol consumption, there was no association between GGT and CVD mortality in men. These results may suggest GGT-specific CVD risks regardless of alcohol drinking, and the different sex results are consistent with our findings.

Furthermore, several epiphenomena are notable for their relationship with GGT and IHD. GGT elevation has been linked to insulin resistance, possibly predicting the risk of diabetes and metabolic syndrome [22,23]. Hepatic steatosis has been proposed as a mechanism for mediating GGT and insulin resistance [24,25], and nonalcoholic fatty liver disease can affect the outcomes of IHD [26]. Our study also showed that the HSI increased according to the GGT quartile. Many studies have investigated the relationship between GGT and cardiovascular disease, starting from the Framingham Offspring Study (FOS), which revealed that increased activity of circulating GGT predicted the onset of metabolic syndrome, the incidence of CVD, and mortality, showing a role for GGT as a marker of metabolic and cardiovascular risk [7]. A recent meta-analysis of 29 cohort studies with 1.23 million participants and 20,406 cardiovascular outcomes revealed an association between GGT and CVD, with an adjusted relative risk of 1.23 (1.16–1.29) per SD higher log GGT [27]. The interaction between GGT and alcohol consumption has long been considered a significant factor in cardiometabolic risk. In a cohort study of 28,838 Finnish individuals, the relationship between GGT and coronary heart disease was more strongly observed in alcohol drinkers than in non-drinkers [28]. In our study, the participants with AST/ALT > 1.5 and ALT ≥ 40 were excluded to minimize alcohol-related liver damage.

Additionally, in a study on Koreans, genetic variables such as rs4820599 may have partially affected the increase in GGT as a factor mediating the risk of diabetes [29]. Finally, animal experiments have revealed that stress conditions can lead to liver inflammation and subsequent hepatic injury, promoting an influx of gut-derived lipopolysaccharides and the increased activation of Kupffer cells in the liver [30]. Stress associations in liver disease have been overlooked, and well-designed prospective studies in humans that address molecular-level mechanisms are warranted.

Koreans comprise a group of East Asians of ethnic homogeneity with lower overall BMI values. GGT elevation is commonly observed without an increase in other liver enzymes among those Koreans who refrain from drinking, and its clinical implications are not well-known. Furthermore, there are very few studies on the relationship between GGT and IHD for non-obese Koreans. Lean Koreans with isolated GGT may be a good target group for evaluating prospective cardiovascular risk, although they have relatively low metabolic risk. Thus, this study can provide public health epidemiological data. Life intervention using mobile devices can be a useful protective measure for those with CVD risk factors in the preclinical stage and is considered an essential topic for future research [31,32].

Some strengths and limitations should be considered and may affect the interpretation of the results of the present study. A major strength of this work was that we investigated a longitudinal cohort study using many Korean individuals linked to HERAS and HIRA data, which are derived from the universal coverage system in Korea. This decreases the chance of missing data. Regarding the data, we excluded those with obesity and diabetes, which are correlated variables that can accompany the cardiovascular disease. This study has several limitations that should also be acknowledged. The study cohort was composed of volunteers sequentially visiting for health examination screenings conducted at a single hospital, and the participants appeared healthier than most community-based cohorts. Thus, the prevalence of IHD in this study is low, which can be a limitation in statistical strength. However, a five-year vascular health assessment has become as important as that over ten years [33,34]. Additionally, we did not consider some comorbidities, such as peripheral artery disease, atrial fibrillation, thyroid diseases, and nonalcoholic fatty liver disease; in addition, a detailed assessment of alcohol consumption was not carried out because these variables were not measured at the beginning of this study. Thus, further studies are warranted to elucidate the longitudinal association between GGT and IHD in consideration of additional history and lifestyle factors. Lastly, the HERAS–HIRA dataset only assessed the newly developed IHD, not the calcium score data or coronary angiography. Thus, further studies are necessary to investigate the clinical and prognostic value of isolated GGT elevation in the Korean population.

## 5. Conclusions

Our study suggests a potential association between elevated isolated GGT levels and future IHD in a large-scale cohort study on lean Koreans without diabetes. In addition, women tended to have a pronounced risk for IHD at high GGT levels. Accordingly, increased GGT levels may be an additional initiative for assessing and managing cardiovascular risk among apparently healthy individuals.

## Figures and Tables

**Table 1:** Baseline characteristics of the study population.

	Q1 (*n* = 2953)	Q2 (*n* = 2187)	Q3 (*n* = 2297)	Q4 (*n* = 2457)	*p*-Value ^1^
Serum GGT (IU/L)	≤14	15–19	20–29	≥30	
Age (years)	42.9 ± 10.1	45.2 ± 10.9	45.9 ± 10.7	46.3 ± 9.6	<0.001
Male sex (%)	14.0	41.6	65.1	80.8	<0.001
Body mass index (kg/m^2^)	21.3 ± 1.9	21.8 ± 1.9	22.4 ± 1.9	22.8 ± 1.7	<0.001
Systolic blood pressure (mmHg)	114.0 ± 13.9	118.4 ± 14.5	121.2 ± 14.3	124.1 ± 13.9	<0.001
Diastolic blood pressure (mmHg)	70.7 ± 9.1	73.7 ± 9.3	75.8 ± 9.3	78.1 ± 9.3	<0.001
Fasting plasma glucose (mg/dL)	87.6 ± 8.3	89.8 ± 9.1	91.1 ± 9.3	93.3 ± 10.0	<0.001
Aspartate aminotransferase (IU/L)	17.5 ± 4.0	18.9 ± 4.4	20.1 ± 4.7	22.0 ± 5.1	<0.001
Alanine aminotransferase (IU/L)	15.0 ± 4.6	17.5 ± 5.5	20.2 ± 6.5	24.1 ± 7.1	<0.001
Total cholesterol (mg/dL)	178.1 ± 31.2	185.6 ± 32.2	189.0 ± 32.5	196.0 ± 34.0	<0.001
Triglyceride (mg/dL)	84.0 ± 38.3	100.0 ± 51.8	116.0 ± 59.6	145.1 ± 87.3	<0.001
HDL cholesterol (mg/dL)	58.0 ± 12.2	55.3 ± 12.5	52.8 ± 12.6	52.7 ± 12.6	<0.001
Log C-reactive protein (mg/L)	−1.1 ± 1.0	−0.7 ± 1.0	−0.5 ± 1.1	−0.2 ± 1.1	<0.001
Current smoker (%)	6.8	17.7	28.9	44.5	<0.001
Alcohol drinking ^2^ (%)	27.1	38.8	47.0	61.1	<0.001
Regular exercise ^3^ (%)	30.3	32.6	31.5	31.0	0.363
HSI score	29.9 ± 2.3	30.4 ± 2.6	31.1 ± 2.8	32.0 ± 2.9	<0.001
Dyslipidemia (%)	22.7	23.6	27.3	38.3	<0.001
Prediabetes (%)	7.8	12.7	15.4	39.9	<0.001
Hypertension (%)	7.8	12.4	17.2	23.7	<0.001

GGT, γ-glutamyltransferase; HDL, high density-lipoprotein; HSI, hepatic steatosis index. ^1^ *p*-values were calculated using one-way ANOVA or Pearson’s chi-squared test. ^2^ Alcohol consumption of ≥140 g of ethanol/week. ^3^ Moderate-intensity physical exercise ≥three times/week.

**Table 2:** Hazard ratios (95% confidence intervals) for the incidence of ischemic heart disease.

		Q1	Q2	Q3	Q4	*p* for Trend
New cases of ischemic heart disease, n		25	38	52	68	
Mean follow-up, years		2.3 ± 1.1	2.3 ± 1.1	2.3 ± 1.1	2.4 ± 1.1	
Pearson–years of follow-up		6821	4975	5366	5886	
Incidence rate/1000 people–years		3.7	7.6	9.7	11.6	
Model 1	HR (95% CI)	1.00 (reference)	1.52 (0.90–2.54)	1.69 (1.02–2.82)	1.93 (1.15–3.21)	0.094
	*p*-value	-	0.115	0.043	0.012	
Model 2	HR (95% CI)	1.00 (reference)	1.68 (0.97–2.92)	1.86 (1.08–3.22)	2.09 (1.20–3.66)	0.075
	*p*-value	-	0.066	0.026	0.009	
Model 3	HR (95% CI)	1.00 (reference)	1.66 (0.95–2.89)	1.82 (1.05–3.16)	1.98 (1.12–3.50)	0.118
	*p*-value	-	0.075	0.033	0.018	

Model 1: adjusted for age, sex, and body mass index. Model 2: adjusted for age, sex, body mass index, smoking status, alcohol consumption, and physical activity. Model 3: adjusted for age, sex, body mass index, smoking status, alcohol consumption, physical activity, mean arterial blood pressure, fasting plasma glucose, and dyslipidemia.

**Table 3:** Hazard ratios (95% confidence intervals) for the incidence of ischemic heart disease according to sex-specific GGT quartile.

**Men**		**Q1** **(≤19)**	**Q2** **(20–26)**	**Q3** **(27–38)**	**Q4** **(≥39)**	***p* for Trend**
Model 1	HR (95% CI)	1.00 (reference)	1.05 (0.62–1.77)	0.98 (0.57–1.67)	1.29 (0.78–2.11)	0.663
	*p*-value	-	0.868	0.931	0.320	
Model 2	HR (95% CI)	1.00 (reference)	1.12 (0.65–1.92)	1.06 (0.61–1.86)	1.31 (0.76–2.24)	0.780
	*p*-value	-	0.680	0.831	0.330	
Model 3	HR (95% CI)	1.00 (reference)	1.11 (0.64–1.90)	1.03 (0.59–1.81)	1.23 (0.71–2.14)	0.8797
	*p*-value	-	0.715	0.922	0.463	
**Women**		**Q1** **(≤12)**	**Q2** **(13–15)**	**Q3** **(16–20)**	**Q4** **(≥21)**	***p* for Trend**
Model 1	HR (95% CI)	1.00 (reference)	1.36 (0.59–3.15)	1.79 (0.80–4.02)	2.38 (1.13–5.01)	0.108
	*p*-value	-	0.475	0.155	0.022	
Model 2	HR (95% CI)	1.00 (reference)	1.73 (0.67–4.47)	2.17 (0.86–5.49)	2.86 (1.19–6.83)	0.110
	*p*-value	-	0.260	0.103	0.018	
Model 3	HR (95% CI)	1.00 (reference)	1.79 (0.69–4.63)	2.18 (0.86–5.54)	2.86 (1.19–6.87)	0.119
	*p*-value	-	0.233	0.101	0.018	

Model 1: adjusted for age and body mass index. Model 2: adjusted for age, body mass index, smoking status, alcohol consumption, and physical activity. Model 3: adjusted for age, body mass index, smoking status, alcohol consumption, physical activity, mean arterial blood pressure, fasting plasma glucose, and dyslipidemia.

## Data Availability

The data underlying this article will be shared upon reasonable request to the corresponding author.

## References

[1] Bai C., Zhang M., Zhang Y., He Y., Dou H., Wang Z., Wang Z., Li Z., Zhang L. (2022). Gamma-Glutamyltransferase Activity (GGT) Is a Long-Sought Biomarker of Redox Status in Blood Circulation: A Retrospective Clinical Study of 44 Types of Human Diseases. Oxid. Med. Cell. Longev..

[2] Takemura K., Board P.G., Koga F. (2021). A Systematic Review of Serum γ-Glutamyltransferase as a Prognostic Biomarker in Patients with Genitourinary Cancer. Antioxidants.

[3] Cho A.R., Kwon Y.J., Lim H.J., Lee H.S., Kim S., Shim J.Y., Lee H.R., Lee Y.J. (2018). Oxidative balance score and serum γ-glutamyltransferase level among Korean adults: A nationwide population-based study. Eur. J. Nutr..

[4] Seo M.S., Lee H.R., Shim J.Y., Kang H.T., Lee Y.J. (2014). Relationship between blood mercury concentrations and serum γ-glutamyltranspeptidase level in Korean adults using data from the 2010 Korean National Health and Nutrition Examination Survey. Clin. Chim. Acta.

[5] Ndrepepa G., Kastrati A. (2016). Gamma-glutamyl transferase and cardiovascular disease. Ann. Transl. Med..

[6] Ferris H., O’Flynn A., Kearney P. (2018). Double trouble: The effect of obesity and alcohol consumption on serum GGT in Irish middle-aged adults. Rev. D’épidémiologie St. Publique.

[7] Lee D.S., Evans J.C., Robins S.J., Wilson P.W., Albano I., Fox C.S., Wang T.J., Benjamin E.J., D’Agostino R.B., Vasan R.S. (2007). Gamma glutamyl transferase and metabolic syndrome, cardiovascular disease, and mortality risk: The Framingham Heart Study. Arterioscler. Thromb. Vasc. Biol..

[8] Sabanayagam C., Shankar A., Li J., Pollard C., Ducatman A. (2009). Serum gamma-glutamyl transferase level and diabetes mellitus among US adults. Eur. J. Epidemiol..

[9] Seo Y., Aonuma K. (2017). Gamma-Glutamyl Transferase as a Risk Biomarker of Cardiovascular Disease—Does It Have Another Face?. Circ. J..

[10] Yoon J., Jung D., Lee Y., Park B. (2021). The Metabolic Score for Insulin Resistance (METS-IR) as a Predictor of Incident Ischemic Heart Disease: A Longitudinal Study among Korean without Diabetes. J. Pers. Med..

[11] Jung D.H., Park B., Lee Y.J. (2022). Longitudinal Effects of Serum Calcium and Phosphate Levels and Their Ratio on Incident Ischemic Heart Disease among Korean Adults. Biomolecules.

[12] Nam G.E., Kim Y.H., Han K., Jung J.H., Rhee E.J., Lee S.S., Kim D.J., Lee K.W., Lee W.Y. (2020). Obesity Fact Sheet in Korea, 2019: Prevalence of Obesity and Abdominal Obesity from 2009 to 2018 and Social Factors. J. Obes. Metab. Syndr..

[13] Jung D.H., Lee Y.J., Park B. (2021). Joint Effect of Hepatic Steatosis and Alanine Aminotransferase Within the Normal Range on Incident Ischemic Heart Disease: A Prospective Study in Koreans. Clin. Interv. Aging.

[14] Sattar N., Forrest E., Preiss D. (2014). Non-alcoholic fatty liver disease. BMJ.

[15] Jung D.H., Lee Y.J., Park B. (2021). Longitudinal Effect of Hemoglobin Concentration with Incident Ischemic Heart Disease According to Hepatic Steatosis Status Among Koreans. Front. Cardiovasc. Med..

[16] Glass G.A., Stark A.A. (1997). Promotion of glutathione-gamma-glutamyl transpeptidase-dependent lipid peroxidation by copper and ceruloplasmin: The requirement for iron and the effects of antioxidants and antioxidant enzymes. Environ. Mol. Mutagen..

[17] Paolicchi A., Minotti G., Tonarelli P., Tongiani R., De Cesare D., Mezzetti A., Dominici S., Comporti M., Pompella A. (1999). Gamma-glutamyl transpeptidase-dependent iron reduction and LDL oxidation–a potential mechanism in atherosclerosis. J. Investig. Med..

[18] Mason J.E., Starke R.D., Van Kirk J.E. (2010). Gamma-glutamyl transferase: A novel cardiovascular risk biomarker. Prev. Cardiol..

[19] Paolicchi A., Emdin M., Ghliozeni E., Ciancia E., Passino C., Popoff G., Pompella A. (2004). Images in cardiovascular medicine. Human atherosclerotic plaques contain gamma-glutamyl transpeptidase enzyme activity. Circulation.

[20] Pucci A., Franzini M., Matteucci M., Ceragioli S., Marconi M., Ferrari M., Passino C., Basolo F., Emdin M., Paolicchi A. (2014). b-Gamma-glutamyltransferase activity in human vulnerable carotid plaques. Atherosclerosis.

[21] Hozawa A., Okamura T., Kadowaki T., Murakami Y., Nakamura K., Hayakawa T., Kita Y., Nakamura Y., Okayama A., Ueshima H. (2007). gamma-Glutamyltransferase predicts cardiovascular death among Japanese women. Atherosclerosis.

[22] Kawamoto R., Kohara K., Tabara Y., Miki T., Otsuka N. (2009). Serum gamma-glutamyl transferase levels are associated with metabolic syndrome in community-dwelling individuals. J. Atheroscler. Thromb..

[23] Lee J.H., Lee H.S., Lee Y.J. (2020). Serum γ-glutamyltransferase as an independent predictor for incident type 2 diabetes in middle-aged and older adults: Findings from the KoGES over 12 years of follow-up. Nutr. Metab. Cardiovasc. Dis..

[24] Thamer C., Tschritter O., Haap M., Shirkavand F., Machann J., Fritsche A., Schick F., Häring H., Stumvoll M. (2005). Elevated serum GGT concentrations predict reduced insulin sensitivity and increased intrahepatic lipids. Horm. Metab. Res..

[25] Grønbaek H., Thomsen K.L., Rungby J., Schmitz O., Vilstrup H. (2008). Role of nonalcoholic fatty liver disease in the development of insulin resistance and diabetes. Expert Rev. Gastroenterol. Hepatol..

[26] Keskin M., Hayıroğlu M.İ., Uzun A.O., Güvenç T.S., Şahin S., Kozan Ö. (2017). Effect of Nonalcoholic Fatty Liver Disease on In-Hospital and Long-Term Outcomes in Patients With ST–Segment Elevation Myocardial Infarction. Am. J. Cardiol..

[27] Kunutsor S.K., Apekey T.A., Khan H. (2014). Liver enzymes and risk of cardiovascular disease in the general population: A meta-analysis of prospective cohort studies. Atherosclerosis.

[28] Lee D.H., Silventoinen K., Hu G., Jacobs D.R., Jousilahti P., Sundvall J., Tuomilehto J. (2006). Serum gamma-glutamyltransferase predicts non-fatal myocardial infarction and fatal coronary heart disease among 28,838 middle-aged men and women. Eur. Heart J..

[29] Lee Y.S., Cho Y., Burgess S., Davey Smith G., Relton C.L., Shin S.Y., Shin M.J. (2016). Serum gamma-glutamyl transferase and risk of type 2 diabetes in the general Korean population: A Mendelian randomization study. Hum. Mol. Genet..

[30] Joung J.Y., Cho J.H., Kim Y.H., Choi S.H., Son C.G. (2019). A literature review for the mechanisms of stress-induced liver injury. Brain Behav..

[31] Tekkeşin A., Hayıroğlu M., Çinier G., Özdemir Y.S., İnan D., Yüksel G., Pay L., Parsova K.E., Vatanoğlu E.G., Şeker M. (2021). Lifestyle intervention using mobile technology and smart devices in patients with high cardiovascular risk: A pragmatic randomised clinical trial. Atherosclerosis.

[32] Hayıroğlu M., Çınar T., Çinier G., Karakaya A., Yıldırım M., Güney B., Öz A., Gündoğmuş P.D., Ösken A., Özkan A. (2021). The effect of 1-year mean step count on the change in the atherosclerotic cardiovascular disease risk calculation in patients with high cardiovascular risk: A sub-study of the LIGHT randomized clinical trial. Kardiol. Pol..

[33] Pylypchuk R., Wells S., Kerr A., Poppe K., Riddell T., Harwood M., Exeter D., Mehta S., Grey C., Wu B.P. (2018). Cardiovascular disease risk prediction equations in 400 000 primary care patients in New Zealand: A derivation and validation study. Lancet.

[34] Panagiotakos D., Pitsavos C., Chrysohoou C., Palliou K., Lentzas I., Skoumas I., Stefanadis C. (2009). Dietary patterns and 5-year incidence of cardiovascular disease: A multivariate analysis of the ATTICA study. Nutr. Metab. Cardiovasc. Dis..

